# Coronavirus disease 2019 (COVID-19) and antibiotic stewardship: Using a systems engineering approach to maintain patient safety

**DOI:** 10.1017/ice.2020.1263

**Published:** 2020-10-12

**Authors:** Julie A. Keating, Linda McKinley, Nasia Safdar

**Affiliations:** 1 William S. Middleton Memorial Veterans’ Hospital, Madison, Wisconsin; 2 Department of Medicine, University of Wisconsin-Madison School of Medicine and Public Health, Madison, Wisconsin

*To the Editor—*In the absence of effective treatments for coronavirus disease 2019 (COVID-19), many hospitalized COVID-19 patients receive antibiotics.^1,2^ Thus far, the literature does not indicate that antibiotics are effective in treating COVID-19, and the incidence of bacterial coinfections appears low.^2^ One analysis reported that while 8% of COVID-19 patients experienced a bacterial or fungal coinfection, 72% of COVID-19 patients received antibiotics.^2^

*Clostridioides difficile* infection (CDI) is associated with broad-spectrum antibiotics frequently used for COVID-19; CDI is thus a significant concern for COVID-19 patients.^3^ Patients at higher risk of severe COVID-19 frequently also have risk factors for CDI such as advanced age and weakened immune systems.^3^ COVID-19 treatments themselves, which often involve extended hospital stays, can also increase a patient’s risk of developing healthcare-associated CDI.^3^ CDI has been identified in patients who received antibiotics as part of their COVID-19 treatment.^4,5^

Given the patient safety risks posed by CDI, effective antibiotic stewardship remains critical throughout the COVID-19 pandemic. However, pandemic-related changes to healthcare delivery (eg, drug shortages, changing pharmacy workflows, and redeployed healthcare workers) have made antibiotic stewardship interventions even more challenging. We present a systems engineering approach to evaluate and modify antibiotic stewardship programs within the constraints of COVID-19 responses, with the overall goal of reducing CDI.

## Systems Engineering Initiative for Patient Safety (SEIPS): A framework to support antibiotic stewardship

Antibiotic stewardship initiatives can involve persuasive (eg, education or audit with feedback) and restrictive (eg, formulary restriction) approaches; these initiatives are used together with appropriate diagnostic and infection prevention measures. These strategies require complex behavioral changes and can involve significant resources such as real-time access to antibiotic stewardship staff for consultation.

The COVID-19 pandemic response has further increased the complexity of antibiotic stewardship.^6^ Prescribers and antibiotic stewardship team members are facing higher and more complex patient loads. The wide-ranging symptoms of COVID-19 may mimic other infections, and a worsening of symptoms is frequently seen 1–2 weeks into the disease that can make it difficult to identify potential coinfections. The length of hospitalization for many patients increases risks of developing healthcare-acquired infections such as ventilator-associated pneumonia that may require additional antibiotic treatment.^7^ These factors, combined with a lack of effective treatment options for severe COVID-19, have resulted in high levels of antibiotic use among COVID-19 inpatients.^1,2^

The structure and effectiveness of an antibiotic stewardship program is dependent on the individual work-system context, including characteristics of the patient population, organizational culture toward antibiotic stewardship, availability of infectious disease and pharmacy personnel, accessibility of clinical decision support tools, and existing policies to support antibiotic stewardship. A systems engineering approach can be used (1) to fully evaluate the roles that work-system elements play in complex antibiotic stewardship interventions and (2) to develop modifications to these elements to support the implementation of interventions. The Systems Engineering Initiative for Patient Safety (SEIPS) provides a framework for this approach. SEIPS defines work-system elements: person(s), technology and tools, environment, tasks, and organization. The interaction of these elements influences care processes and outcomes.^8^

Given the urgent needs to ensure appropriate antibiotic use and reduce CDI risk in COVID-19 patients, a systems engineering approach such as SEIPS can be used to understand the various work-system factors that are involved in antibiotic stewardship and CDI prevention. The interaction of these elements drives antibiotic stewardship, COVID-19 treatment, and CDI prevention processes in each work system; thus, they influence critical patient and organizational outcomes. This SEIPS-based approach provides flexibility for teams to evaluate their own work-system–specific barriers and facilitators to antibiotic stewardship practices within their COVID-19 response.^9^ Teams can then develop strategies to support antibiotic stewardship within the individual work system to optimize patient and organizational outcomes.

## Work system-based elements can be modified to support antibiotic stewardship throughout COVID-19 response

Table 1 lists strategies based on SEIPS work-system elements to support antibiotic stewardship and CDI prevention through COVID-19 responses. The SEIPS framework is Person centered: the COVID-19 patient, prescribers, and antibiotic stewardship team members interact with all work-system elements. People use Technology and Tools (eg, antibiotic prescription decision support tools built into electronic medical records) to enact Tasks (eg, choosing appropriate therapies for the patient, including postponing or de-escalating antibiotics). The Organization’s antibiotic stewardship and infection control infrastructure should continue activities such as reporting antibiotic usage, resistance, and CDI rates. Within the work-system Environment, visual cues reminding prescribers of best practices for antibiotic prescribing in COVID-19 patients may be useful given the high and complex patient loads. Existing CDI prevention practices (eg, environmental cleaning and appropriate diagnostic testing) should remain a priority.

**Table 1.** Practices to Support Antibiotic Stewardship and Reduce CDI in the Care of COVID-19 Patients

SEIPS Element	Antibiotic Stewardship Practice
Person(s)	Avoid antibiotic use unless patient is severely ill or in case of bacterial coinfection
	Prioritize infectious diseases and/or pharmacy availability for antibiotic prescription consultations
	Provide education for staff regarding antibiotic stewardship and COVID-19 treatments
Technology and tools	Develop facility guidelines for antibiotic prescribing to COVID-19 patients
	Build in review periods for updates and evidence-based practices for antibiotic use during COVID-19 treatments
	Build antibiotic prescribing decision support tools into the electronic medical record for COVID-19 patients
	Ensure that virtual communication tools are available as part of workflow for infectious diseases and pharmacy consultations regarding antibiotic prescribing
Organization	Involve antibiotic stewardship team in COVID-19 response and incident command^10^
	Consider joint antibiotic stewardship–infection preventionist team to develop facility’s COVID-19 antibiotic stewardship response
	Regularly review antibiotic use and outcomes in COVID-19 patients and revise facility antibiotic stewardship guidelines accordingly
	Maintain existing antibiotic stewardship programs and practices for patients with and without COVID-19
	Communicate regularly regarding facility guidelines for antibiotic use in COVID-19 patients
	Ensure leadership support and commitment to continued antibiotic stewardship and CDI prevention initiatives
	Include CDI risk as part of rounds for COVID-19 patients
Tasks	Continue to track and report overall antibiotic use and resistance rates as well as antibiotic use specifically in COVID-19 patients
	Track and report CDI rates overall and specifically in COVID-19 patients
	Follow diagnostic and testing guidelines for bacterial and fungal coinfections in COVID-19 patients prior to initiating antibiotic therapy
	Use existing antibiotic stewardship guidelines to treat secondary infections in COVID-19 patients (eg, urinary tract infection)
	Monitor and postpone antibiotic use in stable COVID-19 patients with potential healthcare-acquired infections (eg, ventilator-associated pneumonia)
	Review COVID-19 patient medical history for prior CDI and CDI risk factors when determining treatment plans
	Review and update antibiotic stewardship workflows to account for COVID-19 response-related changes (eg, redeployed personnel)
Environment	Review facility’s pre-existing CDI rates and use rates of CDI-associated antibiotics (eg, fluoroquinolones)
	Monitor antibiotic availability due to pandemic-related shortages and develop alternative therapy plans to avoid use of broad-spectrum antibiotics where possible
	Add visual reminders (eg, signs) about antibiotic use in COVID-19 patients and the need to continue existing CDI prevention practices
	Ensure infection control practices for CDI remain a priority, such as hand hygiene with soap and water, environmental cleaning, and appropriate diagnostic testing

Note. CDI, *Clostridioides difficile* infection.

Research is needed to understand whether, when, and how antibiotics should be used to treat COVID-19 patients while minimizing adverse effects such as CDI. CDI in COVID-19 patients should be investigated to identify risk factors such as use of specific antibiotics, previous CDI, and/or presentation of COVID-19 gastrointestinal symptoms. Research findings should be incorporated into comprehensive evidence-based antibiotic stewardship programs. In the meantime, the urgency of preventing CDI in COVID-19 patients requires adjusting antibiotic stewardship interventions to fit within current COVID-19 protocols. The SEIPS-based approach presented here can help local antibiotic stewardship teams and decision makers adjust existing plans and develop new approaches to support antibiotic stewardship and reduce CDI during the COVID-19 pandemic.
